# Low-Cost COTS GNSS Interference Monitoring, Detection, and Classification System †

**DOI:** 10.3390/s23073452

**Published:** 2023-03-25

**Authors:** Johannes Rossouw van der Merwe, David Contreras Franco, Jonathan Hansen, Tobias Brieger, Tobias Feigl, Felix Ott, Dorsaf Jdidi, Alexander Rügamer, Wolfgang Felber

**Affiliations:** 1 Satellite-Based Positioning Systems Department, Fraunhofer IIS, Nordostpark 84, 90411 Nuremberg, Germanytobias.brieger@iis.fraunhofer.de (T.B.); alexander.ruegamer@iis.fraunhofer.de (A.R.); wolfgang.felber@iis.fraunhofer.de (W.F.); 2 Focal Point Positioning, 1-3 Chesterton Mill, French’s Rd, Cambridge CB4 3NP, UK; 3 Precise Positioning and Analytics Department, Fraunhofer IIS, Nordostpark 84, 90411 Nuremberg, Germany

**Keywords:** global navigation satellite system (GNSS), interference, detection, classification, machine learning, commercial-off-the-shelf (COTS)

## Abstract

Interference signals cause position errors and outages to global navigation satellite system (GNSS) receivers. However, to solve these problems, the interference source must be detected, classified, its purpose determined, and localized to eliminate it. Several interference monitoring solutions exist, but these are expensive, resulting in fewer nodes that may miss spatially sparse interference signals. This article introduces a low-cost commercial-off-the-shelf (COTS) GNSS interference monitoring, detection, and classification receiver. It employs machine learning (ML) on tailored signal pre-processing of the raw signal samples and GNSS measurements to facilitate a generalized, high-performance architecture that does not require human-in-the-loop (HIL) calibration. Therefore, the low-cost receivers with high performance can justify significantly more receivers being deployed, resulting in a significantly higher probability of intercept (POI). The architecture of the monitoring system is described in detail in this article, including an analysis of the energy consumption and optimization. Controlled interference scenarios demonstrate detection and classification capabilities exceeding conventional approaches. The ML results show that accurate and reliable detection and classification are possible with COTS hardware.

## 1. Introduction

**Figure 1:**
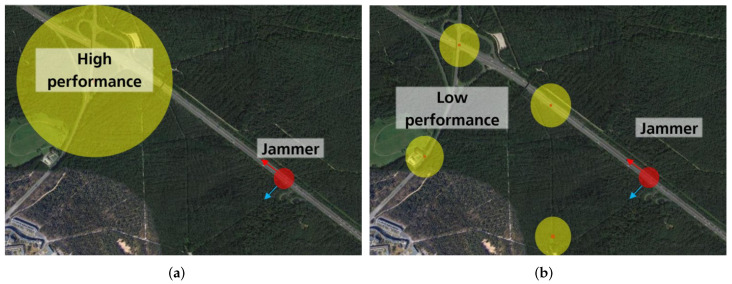
A qualitative example comparison of the symbolic coverage (yellow colored area) of a single high-performance monitoring receiver to a distributed network of low-performance receivers. In both cases, the POI is too low to detect the interference signal (red-colored interference signal), indicating that either the performance must be increased (higher coverage per receiver) or more receivers are required. (**a**) A high-performance interference monitoring system with great sensitivity and a single large coverage area. (**b**) Multiple low-performance interference monitoring systems, each with low sensitivity and small coverage.

Interference signals are a significant problem for global navigation satellite system (GNSS) receivers [1]. Ideally, the signal sources should be located and removed to ensure the reliable operation of GNSS services. However, many interference signals have only a limited local effect and are difficult to detect without monitoring the infrastructure. In addition, the probability of intercept (POI) decreases significantly if the monitors are too sparsely distributed, making it almost impossible to detect, locate, and eliminate sources of interference. Conversely, too many monitoring stations increase the setup, calibration, and maintenance costs. Therefore, a geographic trade-off for interference monitoring networks exists. Figure 1 illustrates that the symbolic coverage of a single high-performance monitoring receiver is insufficient for detecting the jammer (interference source), but a network of low-performance monitoring receivers is also insufficient. It emphasizes that the ideal system has high-performance monitoring (i.e., large coverage per receiver) and a dense network of receivers to maximize the POI.

Several interference monitoring systems exist [2,3,4,5,6,7,8,9,10,11,12,13,14,15,16,17,18,19,20,21,22,23,24,25,26,27,28,29]. There is also an attempt to standardize monitoring approaches [30]. Several systems are developed to be low-cost [3,11,13] or to use software-defined radio (SDR) techniques [3,4,13,15]. However, these systems tend to be expensive purpose-built equipment. As a result, the number of monitoring receivers is too low to be effective. A common strategy is to mount these systems near other critical infrastructures, such as fixed installations on highways [20,24,26], airports [2,18,27,28], or existing geodetic reference networks [6,7,8,25,29], which makes these more effective. A fascinating approach is a monitoring station onboard the international space station (ISS) [21]. Low earth orbit (LEO) satellites cover a larger geographic area to monitor, but are significantly more expensive than current launch costs and may have reduced sensitivity if an interference transmitter antenna suppresses the output signal at higher elevation angles. Therefore, an ideal monitoring system should cover a large geographic area with significantly reduced costs.

There are many different interference waveforms [31,32], and the purpose and intent of the interference signal are determined by knowing what the waveform is. One example is a non-intentional interference from an out-of-band communication signal that could exhibit broadband pulsed orthogonal frequency division multiplexing (OFDM) signals, which should not transmit in the GNSS frequency bands. Therefore, this system should be found, and the manufacturer should implement adequate out-of-band suppression. A second example is an intentional interference (i.e., a jammer) such as a privacy protection device (PPD), which typically emits chirp signals [33]. In this case, it should be found and eliminated as it is illegal in most countries. Lastly, a third example is a distance measurement equipment (DME) signal from an airport, a narrow-band Gaussian pulsed signal that legally coexists in the GNSS bands [34]. These examples highlight the need to detect and have a rudimentary signal classification. Traditionally, signal classification is more complex and resource-intensive than detection, requiring more expensive equipment.

This article showcases a low-cost commercial-off-the-shelf (COTS) interference monitoring receiver with a hardware cost below 200 €, which is at least an order of magnitude cheaper than state-of-the-art interference monitoring systems. Using COTS equipment is not new [35]. However, unlike Gabrielsson et al. [35], the proposed system is significantly cheaper than previous systems. Hence, more receiver nodes are justified in the network as it is considerably more cost-effective. Further, the presented system uses self-calibrating and machine learning (ML) techniques to generalize and limit maintenance and calibration costs. Therefore, the current system improves on several limitations of classical interference monitoring systems. Lastly, the initial results show that the system can accurately and in real-time detect and classify interference signals with performance comparable with traditional methods.

This article extends the conference paper [36] in four ways. First, it presents an extended hardware description of the system, including an analysis of the power consumption, methods to reduce the power consumption, and an investigation of alternative hardware designs. Second, the software design is more detailed, most notably the architectural change of using a Redis database for efficient logging and control. Third, the ML algorithms are explored more deeply to reveal the novel pipeline for interference detection and classification, which facilitates unsupervised label generation to detect and classify unknown interference signals reliably. Fourth, the results of [36] are extended by incorporating more trials to fortify the previous results. Most interesting is the inclusion of tests in a large anechoic chamber with a PPD mounted inside a car for realistic wave propagation effects.

The remainder of this article is structured as follows. Section 2 provides a background to interference monitoring. Section 3 describes the hardware, Section 4 describes the software, and Section 5 describes the algorithms. Section 6 illustrates the test setup, and Section 7 shows the results of the system. Finally, the conclusions are drawn in Section 8.

## 2. Background to Interference Monitoring

This section provides a background for interference detection (Section 2.1), classification (Section 2.2), localization (Section 2.3), and mitigation (Section 2.4).

### 2.1. Detection

The first stage of interference monitoring is to detect interferences. There are several methods to detect interference, and they are grouped into two general categories. First, GNSS receiver outputs monitoring considers the interference’s impact on GNSS processing. Second, the raw in-phase and quadrature-phase (IQ) signal is extracted and often extended to spectrum monitoring to directly measure the interference signals for detection.

Monitoring the GNSS receiver carrier-to-noise density ratio (CN0) is a popular approach to determine the interference impact, as the CN0 reduces when an interference signal is present. The spectral separation coefficient (SSC) equation determines the impact of interference on the CN0 [33,37,38,39]:

(1) $${\left(C/N_{0}\right)}_{\mathrm{eff}}=\frac{1}{\frac{1}{C/N_{0}}+\frac{I/C}{Q_{j}\cdot R_{c}}}$$

where ${\left(C/N_{0}\right)}_{\mathrm{eff}}$ is the CN0 impacted by the interference, $C/N_{0}$ is the interference-free measure, I/C is the interference-to-signal ratio, $R_{c}$ is the chipping rate of the GNSS signal, and $Q_{j}$ is the jamming resistive quality factor of the interference signal. The jamming resistive quality factor $Q_{j}$ is a measure of the interference signal impact and relates to the power spectral density (PSD) of the GNSS signal $S_{s}\left(f\right)$ and the interference signal $S_{i}\left(f\right)$:

(2) $$Q_{j}=\frac{\int_{-\beta /2}^{\beta /2}{∥H(f)∥}^{2}\;S_{s}\left(f\right)\;df}{R_{c}\int_{-\beta /2}^{\beta /2}{∥H(f)∥}^{2}\;S_{s}\left(f\right)\;S_{i}\left(f\right)\;df}$$

where H(f) is the front end response of the receiver, and β is the receiver bandwidth. Equation (1) clearly shows that the GNSS signal strength, the interference signal strength, and the interference waveform significantly impact the CN0. The lower the jamming resistive quality factor $Q_{j}$ is, the more efficient the interference signal is, as a lower interferenceto-signal ratio (ISR) is required for the same degradation.

Simply detecting low CN0 values is invalid, as the CN0 may be suppressed for other reasons, such as signal obstruction (e.g., indoor reception), multipath (i.e., signal distortion), broken equipment (e.g., the antenna is damaged or not connected), or a low-elevation satellite (i.e., significant signal atmospheric and antenna suppression). Valid detection approaches include: comparing the measured CN0 directly with the expected CN0, comparing the CN0 to measurements from other GNSS in the vicinity, comparing the CN0 of the same satellite from different frequency bands (i.e., only one band is interfered with), or comparing the CN0 from different GNSSs (e.g., an interference impacts GPS L1 C/A differently than GLONASS G1). Evaluating the CN0 is a simple post-processing method and can be applied independently of the GNSS receiver hardware. Therefore, it is a popular method to detect interference signals with Android smartphones [40,41,42,43], automatic dependent surveillance-broadcast (ADS-B) flight data [44], or publicly available data [45].

Furthermore, the SSC assumes that the receiver is not in saturation and it is the theoretical degradation of an ideal receiver. If the receiver is saturated, or non-linear effects from the analog frontend are present, the CN0 degrades much faster [39], and ultimately disrupts tracking altogether.

Processing and analyzing the raw received signal is another approach for interference detection. Generally, the signal is processed to obtain some metric to detect [46]:

(3) $$m\left(x[n]\right)\;\overset{H_{1}}{\underset{H_{0}}{⋛}}\;\lambda$$

where $m\left(x[n]\right)$ is an arbitrary metric that depends on the complex digital samples x[n], λ is the detection threshold, $H_{0}$ is the null hypothesis and assumes that the received signal has no interference signal, and $H_{1}$ is the alternative hypothesis and assumes that an interference signal is present. A common approach is to use an energy detector, as a tuned energy detector is the optimal detector for a signal in noise with no a priori information [46] and is a popular choice for interference detection [13,47,48]:

(4) $$m\left(x[n]\right)=e\left[n\right]=T_{s}\sum_{k=0}^{K-1}x\left[n-k\right]x^{*}\left[n-k\right]\;\overset{H_{1}}{\underset{H_{0}}{⋛}}\;\lambda$$

where e[n] is the energy calculated over *K* samples, $T_{s}$ is the sample time, and ${\left(\cdot \right)}^{*}$ is the complex conjugate. A significant issue with this approach is the detector tuning and calibration in practical systems. An implicit energy detection method is monitoring the automatic gain control (AGC) of a GNSS receiver, as it regulates the front end gain based on the received signal energy [43,49]. However, it is risky that the AGC is considered a black box, and unknown effects could deteriorate detection performance.

More advanced detection methods consider spectral techniques [50]. Here, the raw IQ samples are transformed to the spectral domain using a fast Fourier transform (FFT)-based method [50,51], but filter-based approaches such as a spectrum analyzer architecture also exist [52]. Spectrum monitoring approaches are generic, apply to numerous areas beyond GNSS, and have been studied in detail [53,54,55].

The GNSS receiver outputs monitoring and signal spectrum monitoring methods complement each other [13,49,56,57]. For example, GNSS receiver monitoring may yield false detection when a receiver is indoors and has low CN0 values, but spectrum monitoring would reveal no interference. Therefore, a common and advisable approach is to leverage both approaches for improved performance.

### 2.2. Classification

**Figure 2:**
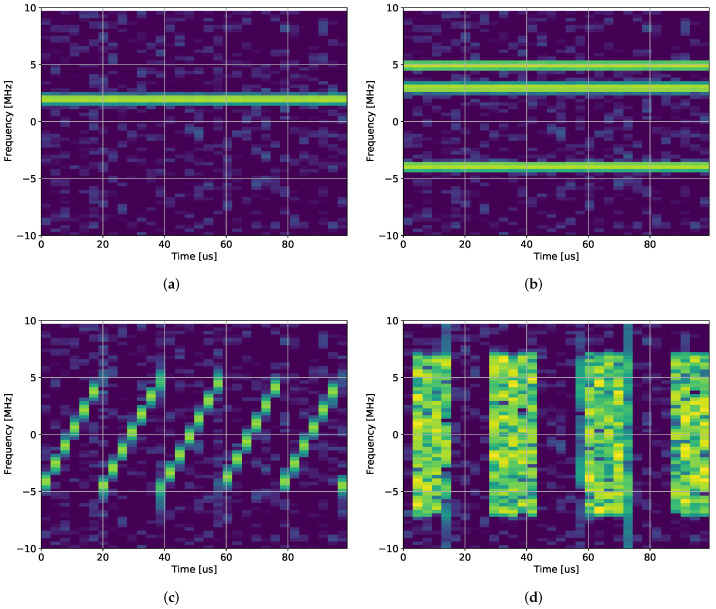
Classification example of different waveform types, showing the spectrograms. Yellow is high power and blue is low power, the signals are: (**a**) Single-tone, (**b**) Multitone, (**c**) Chirp, and (**d**) Pulsed noise.

Interference classification (often called characterization in the GNSS literature [16,19,21]) identifies the interference waveform. If the waveform type is known, the purpose of the signal can be determined, which assists in finding and dealing with the interference signal. Figure 2 shows some examples of different interference signals that can be classified.

Classification is a significantly more complex problem than detection. The traditional approach is to create several purposefully built detectors (i.e., several metrics based on Equation (3)) for each class of signal considered and maximize the correct decision thresholds to distinguish between them [17]. For example, it can be done by using a multiple-hypothesis generalized likelihood ratio test (GLRT). However, it requires significant resources and could be more practical. Hence, a detector tree structure is often deployed to simplify classification. Pre-processing the signal often supports the classification engines. Typical inputs for classification are the spectrogram of the received signal, amplitude statistics, and the signal PSD.

Traditional classification has several limitations: the effort of deriving and tuning optimal detectors, the inability to adapt to new waveforms without new development, high processing costs, and complex logic trees. The latest state-of-the-art techniques employ ML or deep learning (DL) methods to compensate for these weaknesses and to reliably and accurately classify interference signals [36,43,57,58]. Modern ML- and DL-based methods are on par with or even superior to classical techniques in practical situations, as these methods learn non-deterministic and non-linear correlations from data. It improves the classification performance and robustness even in multipath and dynamic cases. In contrast to classical model-driven classification techniques, supervised data-driven approaches implicitly learn these thresholds or informative patterns, enabling accurate classification even near the noise floor [59]. In addition, methods based on artificial intelligence (AI) use exogenous information such as multipath components or other interference sources to map even more specific patterns and specific classes [60]. AI-based methods also implicitly denoise to enable accurate and robust classification [61].

Recent research applied these techniques by adapting support vector machines (SVMs), convolutional neural networks (CNNs) [62], or decision trees (DTs) [63] to the challenges of the GNSS community. The effectiveness of these methods on real data has yet to be determined. Only Voigt et al. [64] compared ResNet18 and time-series (TS) Transformer architectures regarding real-world data classification performance. However, signal properties in the time and frequency domains are similar in the presence and absence of interference signals. Therefore, individually utilizing these methods results in information loss during optimization and negatively impacts the classification accuracy. The performance of ResNet18 and TS-Transformer on data that more accurately reflect the complexities of real-world scenarios, including multipath effects, variable distances and power levels, motion dynamics, and real-world noise, remains to be determined. Based on the current literature, CNNs are a viable approach as they treat the spectrogram of a signal as an image [57,62,65], which facilitates image classification approaches from the computer science literature. Statistical methods incorporating a more diverse range of pre-processed features may benefit using SVMs [66,67]. Recently, autoencoders (AEs) have also proven useful [58,68]. ML has a significant advantage in learning critical information and automatically optimizing the classification process for optimal performance. However, these methods are burdened by several challenges, including expensive representative training datasets and parameter tuning, overfitting risks, and a need for more generalizability.

### 2.3. Localization

Once an interference signal is detected, a permanent solution to stop it is to find and remove it. Therefore, locating interference sources is necessary, and many solutions exist [15,43,49,69,70,71,72]. A popular approach is to collaborate between receivers. The received signal strength (RSS) of the interference signal or the reduction in CN0 (i.e., see the SSC discussion above) are simple measurements used for localization. More advanced systems could use the angle of arrival (AOA) if multiple antennas or a directed receiver antenna are available. Instead, Time difference of arrival (TDOA) is popular, but requires correlation between interference signals, which results in significant ambiguities for periodic signals that limit the applicability for many families of interference signals, such as chirp waveforms. Lastly, frequency difference of arrival (FDoOA) is possible, but is challenging for many waveforms, especially if they are from low-cost imprecise transmitters. For example, broadband indoor localization systems exploit supervised [73] or unsupervised [74] learning strategies for localization (regression of a global position) of user equipment, even in dynamic and multipath situations. Hence, localization is the next expansion of the monitoring system and preliminary performance is presented in this article (see Section 5.1). Therefore, the design considers requirements for localization, such as inter-receiver communication, good timing, and measurement reporting, but in-depth localization analysis is reserved for future research.

### 2.4. Mitigation

A temporary solution to interference signals is to mitigate them through advanced signal conditioning [75,76,77,78]. Interference mitigation methods perform well against some interference signals, but poorly against others. Hence, a single perfect algorithm is unlikely. However, a combination of approaches in a multi-layer approach together with classification yields improved the mitigation capability. By monitoring and classifying the interference signals, the interference threat is quantified. It, in turn, allows a mitigation algorithm designer to focus on the most common interference waveforms, thereby optimizing the mitigation efficiency. Therefore, mitigation algorithm development benefits from interference monitoring. This benefit from surveying is also called electronic support (ES) [53].

## 3. Hardware Design

Section 3.1 presents an overview of the hardware components. Then, Section 3.2 describes the power consumption and optimization cost. Finally, Section 3.3 discusses alternative hardware setups.

### 3.1. Overview

**Figure 3:**
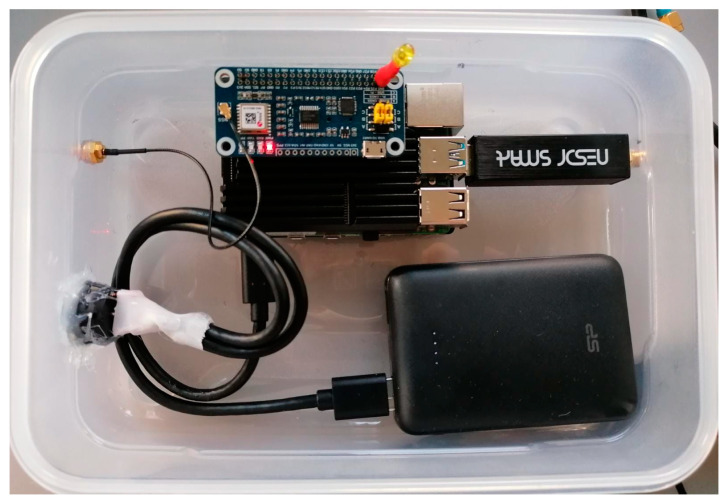
Top-view on the system hardware: the SBC, GNSS pHat, NeSDR, and power bank.

Figure 3 shows a photo of the demonstration version of the monitoring hardware. The central part of the system is a Raspberry Pi model 4B, 4 GB RAM version (Raspberry Pi, Cambridge, UK) single-board computer (SBC), which does the primary processing, interfacing, and networking.

GNSS reception is provided by a GNSS Raspberry Pi hat (pHat) containing a uBlox MAX-M8Q (uBlox, Thalwil, Switzerland). This uBlox receiver can process GPS, Galileo, Beidou, GLONASS, QZSS, and SBAS signals in the L1 band, but only a maximum of three concurrent GNSSs. Therefore, the current system setup configures the receiver to use GPS L1 C/A, Galileo E1 OS-B/C, and GLONASS G1 OS to maximize the spectrum diversity. The SBC connects to the pHat via a universal asynchronous receiver-transmitter (UART) bus over the general purpose input/output (GPIO) interface and decodes the National Marine Electronics Association (NMEA) messages at a 1 Hz rate. The GPS service daemon (GPSD) primarily handles the GNSS receiver. The GNSS pHat can easily be substituted with other GNSS pHats as the pHats uses the standard GPIO interface of the Raspberry Pi. For example, several COTS pHat have dual-band GNSS receivers, such as the uBlox ZED-F9R [79], for a significantly increased cost. In the extreme case, it can even accommodate a geodetic-grade GNSS receiver such as the Septentrio MOSAIC (Septentrio, Leuven, Belgium) [80]. Nevertheless, the current GNSS pHat is ideal for a low-cost design approach.

**Figure 4:**
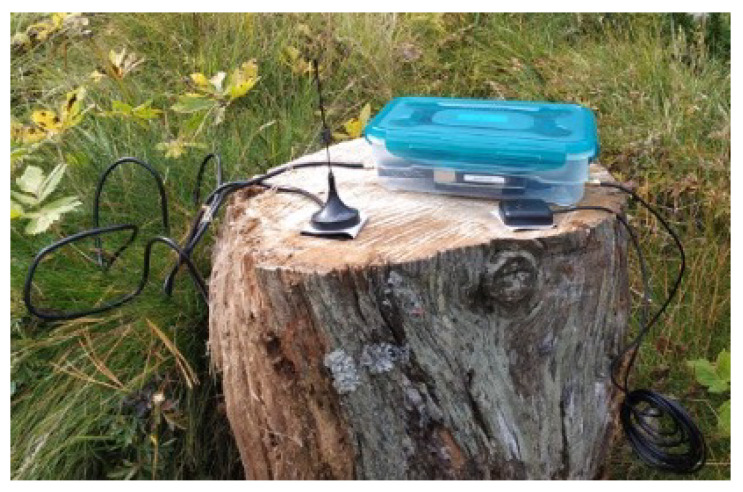
Side view of the system and its antennas in an outdoor setup.

The second sensor is a NeSDR SMArt v4 (Nooelec, Wheatfield, NY, USA) that connects over USB 2.0 and contains an RTL2832U digital video broadcasting (DVB) radio-frequency front-end (RFFE). This sensor is reconfigured as a SDR RFFE and delivers 8-bit complex I/Q samples at a maximum sample rate of 3.2 MHz. It is sufficient to receive narrow-band GNSS signals, such as GPS L1 C/A (1.023 MHz chipping rate), but insufficient to accurately process and localize most GNSS signals. Nevertheless, the bandwidth suffices for detection, monitoring, and classification purposes. The received samples are further processed on the SBC. The GNSS pHat and the NeSDR receivers are supplied with antennas. Hence, no additional antennas are required. The GNSS antenna is a standard low-cost right-hand 24 circular polarized (RHCP) patch antenna with an integrated low-noise amplifier (LNA) and a main lobe towards the sky (zenith). The NeSDR uses a vertically polarized quarter-wave mono-pole antenna (whip antenna). The antenna has a null towards the sky and a main lobe towards the horizon, optimizing for terrestrial interference signals. Figure 4 shows the system outside in the Tupperware enclosure and with the antennas.

The demonstrator requires 6.1 W on average and 7.5 W peak power, including full processing operation. The current concept demonstrator model includes a power bank. It allows for autonomous operation for up to 8 h and is integrated into an ultra-low-cost Tupperware lunch box. Lastly, the Ethernet or the onboard WiFi connects the monitoring system for interfacing. Other connection approaches are discussed in Section 3.3.

### 3.2. Power Consumption and Optimization

A Joy-IT TC66C USB power meter (Joy-IT, Neukirchen-Vluyn, Germany) measures the power consumed by the interference monitoring system with different configurations. The system is switched on, and a minimum of 5 min is waited before conducting any tests to allow the system to stabilize. Next, the average power and peak power over 60 min is measured for each configuration. This approach is rudimentary and has significant tolerances, but it facilitates a rough idea of the power consumption of the different stages. Table 1 shows the various configurations and the measured power profiles.

All hardware is connected in the “Full” profiles (Profiles 1 to 4), but different interfacing and late-stage processing are switched on or off. These include switching the web service for easy interfacing off (Profiles 2 to 4), switching communications and networking off (Profiles 3 and 4), or disabling the initial ML detection (Profile 4). In the “Only GNSS” configuration (Profile 5), the NeSDR is removed, using less hardware, and in the “SDR” configurations (Profiles 6 and 7), the GNSS pHat is removed. In the “Only SDR” configuration, the NesDR is connected, data are recorded, and the spectral processing (see the software and algorithmic design sections) is switched on. The spectral processing is switched off in the “No SDR proc” configuration. Finally, in the last setup (Profile 8), the GNSS pHat and the NeSDR are removed, and the Raspberry Pi is tested in idle mode. From these measurements, the following deductions are made:

- (1) minus (2): Power of web server processes (expectation: low power) = 21 mW;
- (2) minus (3): Power of WiFi and networking (expectation: medium power) = 394 mW;
- (3) minus (4): Power of ML inference processing (expectation: low power) = 99 mW;
- (5) minus (8): Power of the GNSS receiver with LNA and logging (expectation: medium power) = 463 mW;
- (6) minus (7): Power of SDR FFT features processing (expectation: high power) = 1.597 W;
- (7) minus (8): Power of SDR interface (expectation: medium power) = 1.473 W;
- (8): Raspberry Pi idle and hardware overhead (expectation: high power) = 2.219 W.

**Figure 5:**
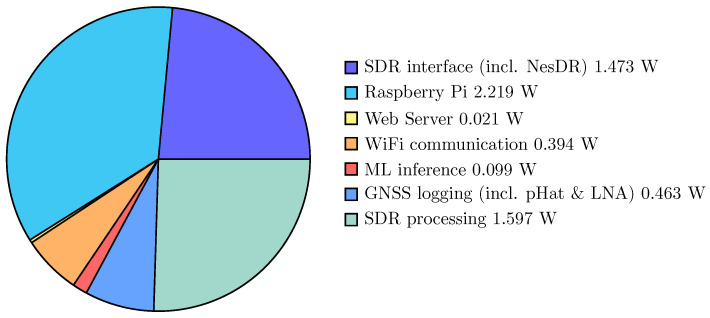
Average power consumption of the different parts of the monitoring station, in [W].

Figure 5 shows the power distribution. The most significant part, roughly one-third of the power, is contributed to the Raspberry Pi hardware in idle mode. It indicates that more energy-efficient solutions than the Raspberry Pi 4B for a monitoring station can be considered as a first optimization step. Nevertheless, a SBC still consumes considerably less power than other monitoring stations based on fully-fledged computers. Choosing a different SBC that is more power efficient may reduce this part of the energy consumption. However, this could limit the rapid prototyping that the Raspberry Pi allows with its large community and associated hardware and software resources and may limit the processing capabilities it supplies for the SDR processing.

The second largest part is the power consumption for the SDR processing. The SDR processing mainly consists of FFT and statistics calculation, which are parallelized efficiently. Therefore, a central processing unit (CPU) may not be the best processing platform. A possible improvement of the system is to replace the Raspberry Pi 4B with a more power-efficient SBC, such as a Raspberry Pi Zero 2 W (according to [81], it has half the power consumption in idle mode), and to use an efficient co-processor for the SDR operations such as a field-programmable gate array (FPGA) [82], a dedicated digital signal processor (DSP) [83], a graphics processing unit (GPU) [84], or tensor processing unit (TPU) [85,86]. The core concept is to leverage the strengths of different architectures for efficient processing [87]. However, all such approaches require significant hardware and software redesign, which results in higher development costs. Nevertheless, for energy efficiency and large-scale deployment, such optimizations are crucial.

Another approach is to change the architecture to minimize the SDR processing. A simple approach is to transmit the raw IQ data from the SDR to a server for processing, thereby omitting the SDR processing on the monitoring node altogether. However, it requires more data to be transmitted over the communication link, resulting in more power consumption from communication (e.g., WiFi transmission), higher data throughput (i.e., it is a burden on the network), and, if an external communication link is used (e.g., mobile networks using LTE or 5G), considerably more costs. The benefit is that it may reduce power consumption on the receiver and facilitate higher fidelity classification algorithms on the server. Given these aspects, such an inefficient system motivates processing on the SBC to limit data throughput.

Alternatively, the SDR processing is only applied when an interference signal is expected. In such an architecture, the SDR is switched off by default, and the initial interference detection is based only on the GNSS pHat measurements. Once an interference signal is detected, then the SDR processing is enabled for improved detection and interference classification. It could potentially save 25% of the power consumption in the default mode. However, this approach limits performance to only the GNSS receiver and applies to interference signals that are present longer. An interference signal only present for a short duration will not be recorded if there is a delay in enabling the SDR processing. An example is a roadside monitoring station to detect PPDs in passing vehicles: a vehicle may only be observed for a second or two while traveling on a highway. With the 1 Hz update rate of the GNSS receiver (or the worst case 5 s delay from ML detection, see Section 4), it may be too slow to realize that an interference signal is present and switch the SDR processing on. Therefore, dynamically enabling the SDR may save power, but has limitations for several applications.

This concept may also be extended by periodically switching the entire platform off or into sleep mode. An application could also be the roadside monitoring case, where the interference monitoring station is only online in peak traffic. It would improve the number of vehicles observed to the system power consumption ratio, resulting in an efficient engineering solution. However, it significantly reduces the POI, which reduces system coverage.

The third largest part is the power used by the NeSDR RFFE. It shows that the idle hardware power requirements should not be underestimated. A possible improvement is to select a more power-efficient SDR RFFE, but it is challenging to find an RFFE that is both power-efficient and low-cost, such as the NeSDR. However, suppose the co-processor idea of the previous paragraph is combined with using a different RFFE. In that case, other SDR RFFEs that already includes an FPGA, e.g., the Lime SDR mini (Lime Microsystems, Guildford, UK, https://limemicro.com/products/boards/limesdr-mini/, accessed on 23 March 2023), could be a more efficient approach.

The remainder of the power consumption, about one-sixth, is used for the GNSS receiver, ML inference, WiFi communication, and running the web server. It shows that the usual arguments that communications (i.e., WiFi) and ML are issues for power efficiency are not valid in this case. The recorded data were sent once per minute in the test configuration, which is a fairly low rate. However, as most of the data are pre-processed with spectral features, the data upload requirements of the interference monitoring station are low (see the software and algorithmic Section 4 and Section 5). Nevertheless, other more power-efficient communication protocols could be considered. Alternatively, the upload rate can be optimized to limit power consumption and data throughput. For example, only when the initial detection raises, is the alarm data sent to the server. However, as the WiFi power consumption is insignificant to other requirements on the system, such optimizations have lower priority.

The shortest day of the year in Nuremberg, Germany, is slightly longer than 8 h, and the mean power consumption of the monitoring station is slightly below 6 W. This indicates that at least an 18 W solar panel is required for fully autonomous operation over 24 h. Accounting for power conversion losses, battery losses in charging and discharging, and sub-optimal panel orientation losses, a standard 50 W solar panel with a battery system suffices (i.e., accepting a 36% efficient system). With current technologies, such a solar panel would require a surface area of approximately 0.64 m${}^{2}$ and cost about EUR 200.

Alternatively, if the system runs from the power grid, the yearly power costs assuming the current 0.30 €/kWh costs in Germany is:

(5) $$\begin{array}{cc} \mathrm{Yearly}\;\mathrm{Energy}\;\mathrm{Cos}t & =365\;\mathrm{days}\times 24\;h\times 0.30\;€/\mathrm{kWh}\times 6.107\cdot 10^{-3}\;\mathrm{kW} \end{array}$$

(6) $$\begin{array}{cc} & =16.05\;€ \end{array}$$

Both solar panel and yearly operational energy costs are relatively low, indicating low operating costs compared to hardware costs. However, if presented with the option, using power from the power grid is better. It has lower upfront costs, requires less maintenance (e.g., replacing batteries or cleaning the solar panels), is more reliable, and is less prone to theft. Furthermore, this analysis provides an initial expectation for the final deployment.

### 3.3. Alternative Hardware Setups

A large selection of COTS components can improve some aspects of the monitoring node. These alter the cost, energy consumption, or performance. Some improvements are considered in this section.

Due to the current Raspberry Pi shortage [88], the selected SBC is challenging to obtain—especially at a reasonable price. There are many COTS SBC alternatives [89], but these often do not have the large Raspberry Pi community and abundant resources that facilitate rapid prototyping. However, the other communities and resources could improve in the future.

**Figure 6:**
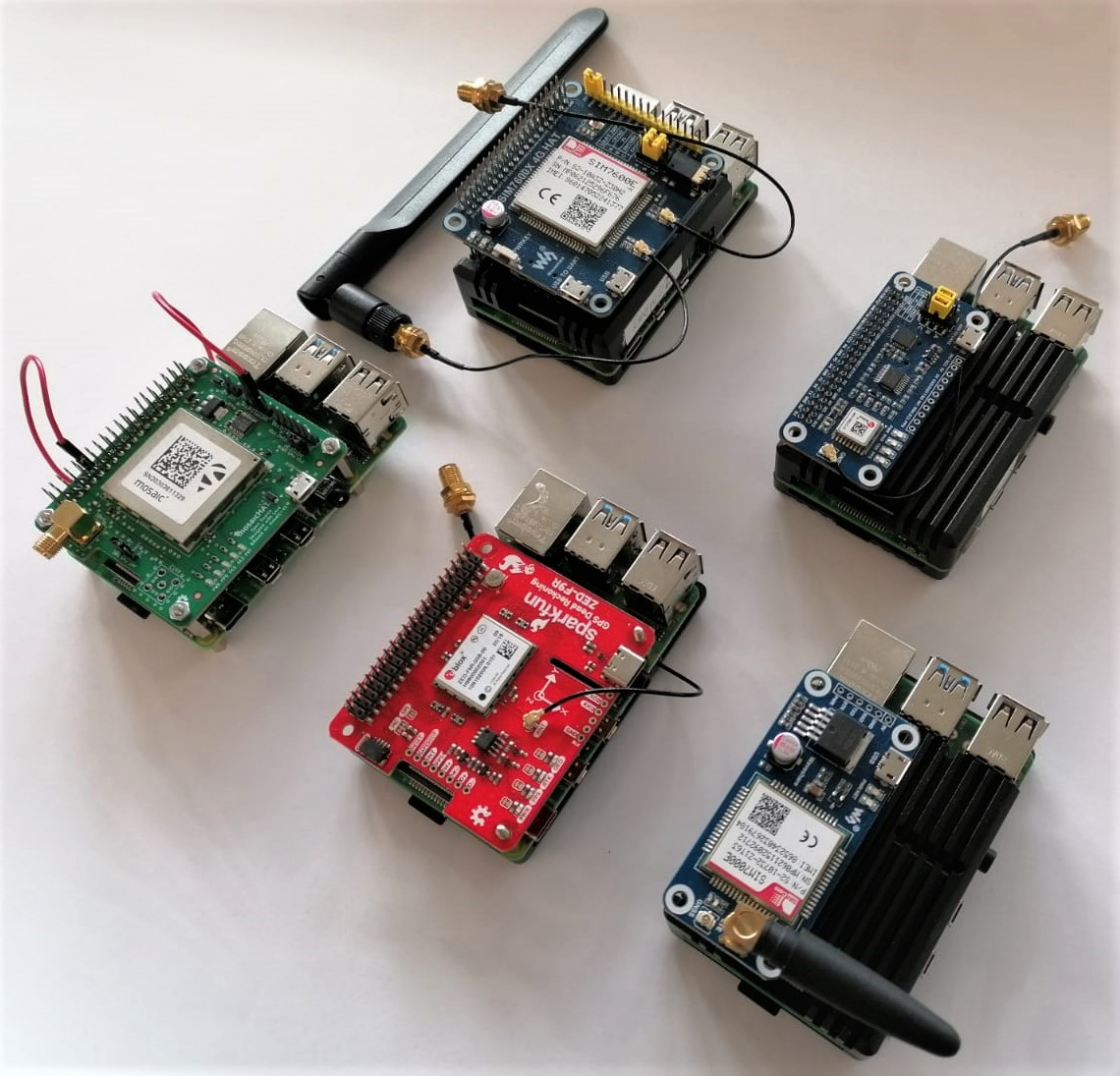
Alternative hardware designs. The top left is a high bandwidth LTE and GNSS pHat. Top right is a low-cost GNSS pHat with a uBlox MAX-M8Q. Bottom left Septentrio MOSAIC pHat. Bottom center uBlox F9R pHat. Bottom right NB LTE and GNSS pHat.

Many GNSS pHats could be used, as described in the previous section. Many of these provide superior performance (especially when multi-band GNSS is used), but are more expensive. Therefore, a cost-to-performance trade-off exists. Other pHats also combine GNSS reception with mobile communications, such as LTE. Figure 6 shows several pHats used for different prototyping approaches in the monitoring station.

The default communication protocol in the monitoring station is WiFi, which is practical and straightforward for small-scale deployments and tests. Furthermore, WiFi is already available on the SBC, so no extra components are required. Currently, evaluations to replace the WiFi communication are in process. However, several other communication protocols are considered for the monitoring station. Mobile communications, such as LTE or 5G, benefit in that the monitoring station only needs a connection to the mobile network. It allows the platform to be deployed anywhere it has access to a network, and modern mobile networks are ubiquitous, but some dead zones often occur. The limitation is that an extensive network of monitoring nodes can incur high costs over the mobile network for data transmission, increasing operational costs. For example, the current monitoring system requires about 3.3 GByte/month (approximately 10.2 kBit/s) to report all data, which costs 10 € per month [90], or 120 € per year. Comparing these costs to Equation (6) demonstrates that the network costs are considerably more than the power costs. Limiting how often data are sent could minimize these costs. Another approach is integrating the GNSS monitoring into a mobile network, such as 5G, as this could create a beneficial interdependency between the network and the monitoring stations [91].

Tests with pHats with combined Long-Term Evolution (LTE) and GNSS modules have been done. LTE integration in the pHats yields the significant challenge that the GNSS NMEA protocol and the LTE data have to share the same serial connection. Sharing the same serial link significantly increases the software development effort, as additional care must be taken to identify each data byte as part of the GNSS or LTE data. Hence, an extra LTE USB stick is used to lower the development complexity. This aspect is still under further development.

Low-throughput long-range protocols are applicable, such as LPWAN, LoRa, RPMA, MIOTY, or DASH7 [92]. These offer larger coverage areas, lower power consumption, the possibility to self-administrate, and an abundance of open networks (e.g., The Things Network (https://www.thethingsnetwork.org/, accessed on 23 March 2023) or LoRa-Alliance (https://lora-alliance.org/, accessed on 23 March 2023). However, they have significantly lower throughput rates, which is not a limitation of the current monitoring system requirements.

Lastly, other SDR RFFEs can be used. The NeSDR uses comparatively high power, has small receiver bandwidth, limited dynamic range (8-bit IQ), unstable clocking, and has no processor on board (e.g., FPGA for signal conditioning), but it is low-cost. A classic and popular board is the HackRF One (Great Scott Gadgets, Lakewood, CO, USA, https://greatscottgadgets.com/hackrf/one/, accessed on 23 March 2023), with an improved sample rate of 20 MHz, but it is larger and more expensive than the NeSDR. The CaribouLite, Haifa, Israel (CaribouLabs, https://www.crowdsupply.com/cariboulabs/cariboulite-rpi-hat, accessed on 23 March 2023) is a newer board with a pHat, making it convenient. It also has an onboard FPGA for basic signal processing. Therefore, it is an exciting new board that could make the interference station more compact and streamlined. The LimeSDR Mini (Lime Microsystems, Guildford, UK, https://limemicro.com/products/boards/limesdr-mini/, accessed on 23 March 2023) has more dynamic range with 12 bits, a receiver bandwidth of 40 MHz, and a larger FPGA. It has superior performance, but is also more expensive. These three boards are interesting candidates for improving the system, but there are many other COTS SDR RFFEs [93]. An RFFE with more dynamic range, larger bandwidth, or better clocking stability leads to superior detection and classification capabilities or combining it with other sensors [57,94].

## 4. Software Design

**Figure 7:**
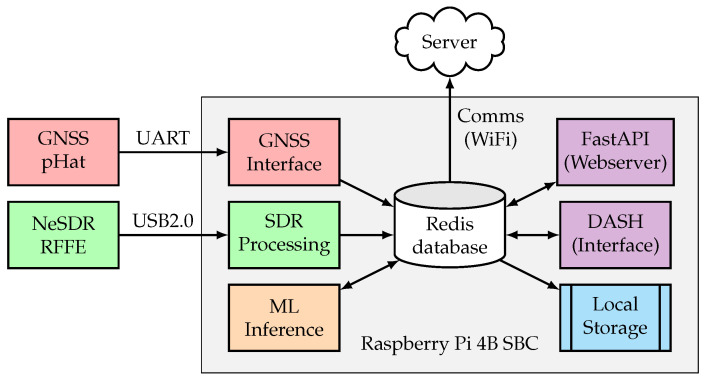
Software flow diagram and interconnections.

The software architecture changed from an integrated system to multiple micro-services connected via persistent storage to allow modularity, expandability, and robustness. All the components are connected to a Redis data structure server, where data are stored for logging or configuration purposes. The updated structure in Figure 7 consists of six major modules: the GNSS receiver interface and data extraction (red), the NeSDR RFFE receiver interface and SDR pre-processing (green), the ML detection model (orange), the data logging and uploading infrastructure (blue), the web application programming interface (API) and interface (purple), and in the Redis database that binds all the components (white). All the modules were implemented in Python 3.8 and executed as services with “systemd”.

The GNSS interface connects to the GNSS pHat via the Unix-based GPSD service. GPSD interfaces the GNSS pHat, decodes the NMEA messages, and extracts the GNSS measurements, including the receiver fix status, the position, velocity, and time (PVT) solution, the expected PVT error, the number of satellites in tracking and used for PVT, the dilution of precision (DOP) of the current constellation, the pseudo-random noise (PRN) code of the satellites in track, the CN0 of each satellite, the satellite elevation, and azimuth. The values are parsed and saved in the Redis database as they arrive from the GNSS pHat. The NMEA messages are configured at a 1 Hz update rate.

The SDR processing connects to the NeSDR and records data with a center frequency of 1575.42 MHz, a sample rate of 2.56 MHz (higher sample rates resulted in instabilities with recording, such as dropped samples), and 8-bit I/Q data. For efficient batch processing, the data are segmented into 1 s snapshots. The snapshots are passed through a moving average (MA) DC-blocker and de-interleaved with a polyphase quadrature filter (PQF) into 64 channels with 40 kHz bandwidth. The PQF block (*pfb_channelizer_cc*) in the current implementation is the same as a windowed short-time Fourier transform (STFT), but it is implemented more efficiently in the GNU Radio architecture. The SDR processing utilizes GNU Radio for the signal processing components. The energy and kurtosis statistics of each channel and the spectral entropy over all channels are determined. Recently, similar pre-processing approaches for interference classification in GNSS have been proposed [67,95]. The gain calibration of the RFFE for the correct gain settings is still challenging. Thus, the current model performs a software auto-calibration when the monitoring station ensures no interference signals are present through a feedback loop from the ML detection. Finally, the spectral features are saved in the Redis database at 1 Hz (i.e., synchronized to the batches).

A local ML interference detection inference runs in real-time using a buffer of 5 s (i.e., a delay of up to 5 s may occur) of the spectral features recorded from the SDR and realizing a detection prediction at 1 Hz. The ML consists of a histogram-based gradient boosting classification tree trained on a measurement campaign (see Section 6) and has shown good detection results previously [96]. The prediction is sent to Redis to log and activate hardware alarms in the form of a LED and a buzzer.

A separate micro-service is responsible for collecting the relevant data from the Redis data structure server, saving it locally for long-term storage, and uploading it to the external server at 1 min intervals. The latter is done for further ML classification inference with more computationally demanding models and to enable collaborative detection and interference localization. The uncompressed data rate is low (about 4.6 MByte/h, or 3.3 GByte/month). An adequate data compression (to a few characteristic features) reduces the data throughput by a factor of about four.

**Figure 8:**
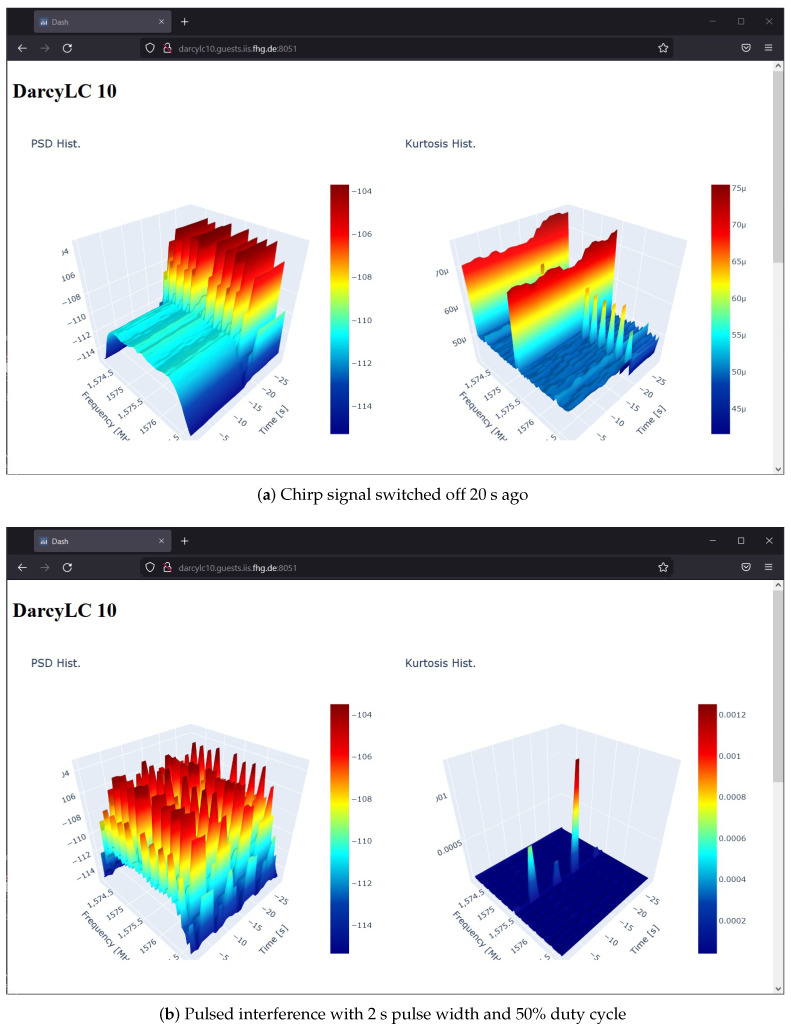
Screenshot of the web server front end, with interference visualizations of the spectral power and spectral kurtosis. The real-time measurements are done in a controlled laboratory.

An essential feature of the updated version is the web server, which includes an interface, data extraction, and configuration. The interface allows real-time visualization of the spectral features. The API provides access to the local long-term storage data and to modify the device configuration. The configuration allows flexibility per device to change the rates at which data are recorded locally and uploaded, the satellite constellations being monitored, the local ML model used for detection, and the spectral processing configuration such as the center frequency, the frequency resolution, and the sample rate. Figure 8 shows a screenshot of the server front end with real-time interference visualization.

**Figure 9:**
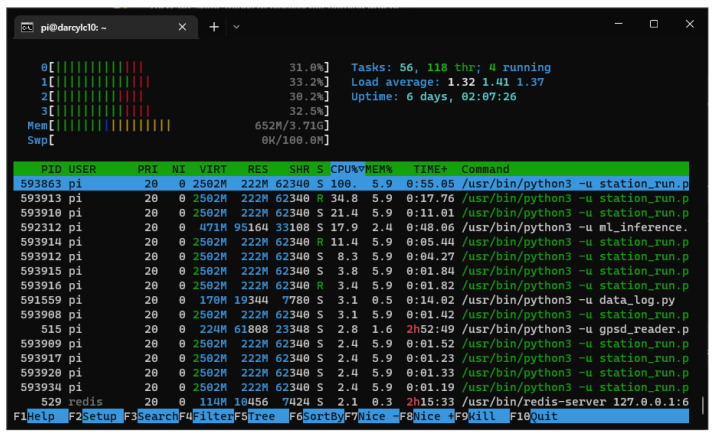
System usage with all modules running according to Profile 1.

In addition to the main modules, system utilization is recorded for device health monitoring and automatic restarts in case of failures. An example system utilization with all processing enabled is shown in Figure 9. On average, the internal system health monitoring measured a CPU utilization of 31.8% and 645 MB of random access memory (RAM). It indicates that the Raspberry Pi is can handle the whole system without issue.

The GNSS pHat is used for accurate timing of the system. It allows millisecond precision synchronization among multiple device recordings. Each sensor nodes synchronizes to the sensor network using the GNSS time from the NMEA messages. It is achieved with the with the 1-pulse per second (PPS) signal form the GNSS pHat over the GPIO of the SBC.

Lastly, a significant improvement in the software is to employ the 64-bit instruction set, which was released in Q1 2022 [97]. A noticeable improvement in processing efficiency for the SDR processing in GNU Radio was observed. It led to using a finer 64-channel FFT for the spectral processing instead of the older 32-channel FFT.

## 5. Algorithmic Design

Compared with the original implementation [36], the ML pipeline is slightly tuned through experience with realistic experiments [43,57,58,94]. It is now more adequate for handling different data input types, i.e., time series such as C/N0, AGC, and elevation and images of spectrograms.

Since the framework focuses on the detection and classification of GNSS interference signals, the pipeline is designed following four steps:

1. Pre-processing,
2. Critical snapshot detection,
3. Classification of interference types,
4. Post-processing based on the uncertainty of estimates.

Section 5.1 first introduces the concept of our supervised ML pipeline before Section 5.2 and Section 5.3 describe its pre-processing and ML-based detection and classification components. Section 5.4 describes the mechanic that ensures the reliability of the pipeline with uncertainty estimation.

### 5.1. Pipeline

**Figure 10:**
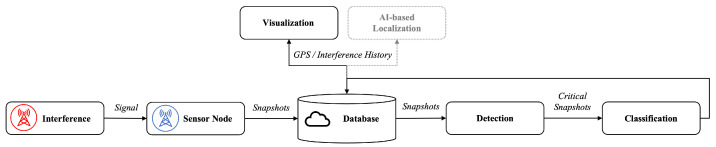
The ML-based pipeline detects, classifies, and visualizes potentially interfered GNSS signals. The boxes (outlined in black) represent all relevant pipeline components, and the iterative text on the edges represents the output/input of each component. Information flows from left to right. A database in the cloud synchronizes all information and thus provides the history of all events for downstream visualization and localization.

Figure 10 depicts the processing pipeline. The supervised pipeline was first introduced by van der Merwe et al. [36] and Brieger et al. [57]. Data flows from the left (input to the model) to the right (predictions of the model). The listeners (i.e., the sensor nodes) receive the signals (with or without interference) in real-time. The signals are collected, synchronized, and stored (i.e., snapshots from multiple listeners) in the database in the cloud.

The multi-stage framework pre-processes features from different (multi-modal) input streams and fuses them (Section 5.2). Entropy, kurtosis, CN0, AGC, and elevation features are extracted from data from deterministic and realistic measurement campaigns. High-rate (1.0 Hz) sampled or interpolated snapshots of both spatial features and time series data thereof are fed to the detection and classification components as a spectrogram (i.e., image) or as time series (i.e., array). From there, the database pushes new signals to the detection component. The classification component further categorizes only the detected interference signals.

In a post-processing step, Monte Carlo dropout (MCD) [98] is applied to the fused components to assess the uncertainty of each estimate (see Section 5.4). The $F_{\beta =2}$ score evaluates the performance and efficiency of the framework (see Section 5.4). Next, a detection event is stored (states: interference and no interference), along with the category, the uncertainty values for both the detection and each category, and a compressed version of samples used in the database.

From there, the database handles requests for the visualization component (see Section 5.4.1). The graphical user interface (GUI) visualizes selected sensor nodes at known locations (see the blue antenna symbols in Figure 10), and the appearance of interference signals (see the red antenna symbol), when a listener detects an interference. The GUI also handles the history of events, thus visualizing a heat-map of interference signals along fixed deterministic trajectories (roads). Red indicates a high amount of detected interference, and the green represents no detected interference. For details on the visualization component, see Section 5.4.1.

### 5.2. Pre-Processing

Individual pre-processing steps are performed for each method, to prepare the data in two different formats.

#### 5.2.1. Data Pre-Processing for Random Forest

In the pre-processing stage for random forest (RF), statistical features are extracted from the IQ data obtained from the SDR. It includes 64 channels for kurtosis and 64 channels for Fourier coefficients. Information such as the latitude, longitude, and the number of available and utilized satellites, is obtained from the GNSS receiver. Analyzing the feature importance reduced the number of recorded features from 310 down to 36. Each feature is normalized through individual scaling using Equation (7).

(7) $$\mathrm{Normalization}:x^{\prime }=\frac{x-\min(x)}{\max(x)-\min(x)}$$

#### 5.2.2. Data Pre-Processing for ResNet

**Figure 11:**
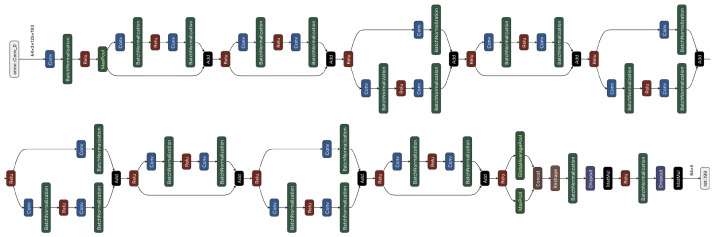
Overview of the ResNet18 architecture. Note that data flows from left to right and from top to bottom. The colored boxes represent the following components: blue = *Convolutional layer*, red = *ReLU layer*, green = *Batch Normalization* or *Max Pooling layer*, and white = *Input/Output layer*.

**Figure 12:**
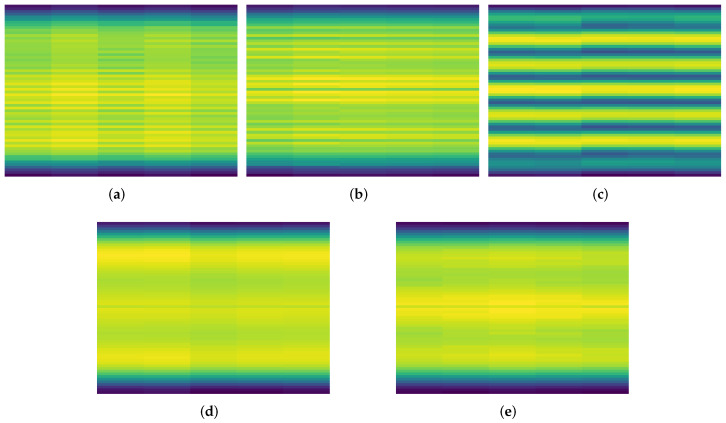
Spectrograms of 5 s of signals, yellow is high power and blue is low power. The signals of the interference signals are 10 to 30 MHz wide, but the sensor node records only a bandwidth of 2.56 MHz so it can see a small portion of the signal. The signals are labeled as Interference signal type 1 to type 4 (**a**–**d**), and None (signal without interference signals) (**e**).

In the pre-processing stage for ResNet, only the 64 channels for Fourier coefficients are extracted from the raw IQ data obtained from the SDR. Every 5 s of these data are plotted as a spectrogram, so there is no overlap between two sequential spectrograms. These 193×125 RGB images are saved as a portable network graphic (PNG) files. So the input tensor for network is 3×125×193 as seen in Figure 11. There is a 50% chance that they are flipped horizontally to increase variation. Figure 12 shows some examples of the PNG files for selected interference signals.

### 5.3. Processing

The features are passed to the main processing components after the data are pre-processed. The main processing components consist of detection and classification mechanisms. Both the detection and classification tasks are formulated as supervised learning problems, with the detection problem further defined as a binary classification task.

#### 5.3.1. Detection vs. Classification

The detection problem is formulated as a binary classification problem. Hence, the supervised detection of an anomaly in a set of features employs the same architectures that are employed for the supervised classification task. Consequently, Section 5.3.2 describes the architectures of the AI models that are employed for both detection and classification tasks. However, before introducing the architectures, the detection and classification tasks are presented.

The first stage of interference monitoring is to detect a interference signal. There are several methods to detect interference, and they are grouped into two categories. First, GNSS receiver output monitoring considers the interference impact on GNSS processing. Second, signal spectrum monitoring considers the raw received signal samples. Therefore, a great approach is to leverage both strategies for improved performance. Consequently, supervised learning techniques are used to exploit the most relevant information from features to optimize the process of interference detection and classification.

Traditional classification has several limitations: the effort of deriving and tuning optimal detectors, the inability to adapt to new waveforms without new development, high processing costs, and complex logic trees. CNNss are a good approach as they consider the signal spectrogram as an image [62], which facilitates image classification approaches from the computer science literature.

The categorization process is developed as a supervised multi-class classification task that returns the interference type and the uncertainty of the prediction, i.e., mean and variance. A RF, proposed by Breiman et al. [99], is deployed for this task. It is a classifier containing multiple DTs, and voting returns the final result. The RF developed and optimized in the presented pipeline efficiently provides the most accurate classification results and the corresponding uncertainty.

The pessimistic metric $F_{\beta =2}$ [100] was used to evaluate the results. This metric weights recall and precision, which measures the errors related to the positive class, to give false-negative estimates more importance, with β being the relative importance of recall over precision. The maximum score is 1.0, which indicates an accurate classification.

(8) $$F_{\beta =2}=\frac{\left(1+\beta^{2}\right)\mathrm{TP}}{\left(1+\beta^{2}\right)\mathrm{TP}+\beta^{2}\mathrm{FN}+\mathrm{FP}}=\frac{5\mathrm{TP}}{5\mathrm{TP}+4\mathrm{FN}+\mathrm{FP}}$$

where TP is the true positive, FN is the false negative, and FP is the false positive. This metric penalizes missed outlier detections (i.e., FN), which is crucial for interference detection.

#### 5.3.2. Architectures

Two distinct architectures classify the data in two different input formats. A straightforward ML method, RF, classifies the time-series data, i.e., CN0, AGC, and elevation. In contrast, a complex deep learning approach using the ResNet18 architecture classifies the spectrogram images.

RF is a ML algorithm that utilizes an ensemble of decision trees to make predictions. The algorithm builds multiple decision trees on randomly selected subsets of the training data and features. Each tree in the forest makes a prediction, and the final prediction is the aggregate of the predictions from all the trees. RF is a popular and powerful algorithm that effectively handles high-dimensional datasets and is less prone to overfitting.

**Figure 13:**
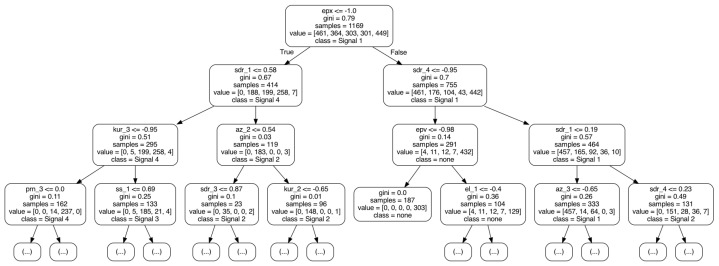
Example tree of a trained RF model. Dependent on the properties of the current features, the RF model (at each block) makes a decision that is true (left arrow) or false (right arrow) until the leaf of the RF model is reached and a decision for classification is made.

An RF consisting of 1000 DTs is selected for classification. *Gini impurity* criterion is applied, and the square root of the number of features for each split is selected (The Random Forest is configured as follows: criterion = ‘gini’, max_depthint = None, min_samples_split = 2, min_samples_leaf = 1, min_weight_fraction_leaf = 0.0, max_features = ‘sqrt’, max_leaf_nodes = None, min_impurity_decrease = 0.0). Figure 13 shows an example of a single DT in the RF model.

ResNet (the most prominent residual learning framework) enables the training of deep networks [101]. The trick with residual networks is that the gradient is routed much deeper into the network, making it more durable, and the gradient does not vanish as quickly. This characteristic and its unique architecture also enable the extraction of temporal information when a sequence of features is processed. Conversely, essential information remains deep in the network and can be used profitably by deeper layers. Hence, residual networks are easier to optimize and can gain accuracy with a greater depth. A residual network that was pre-trained on the ImageNet dataset (https://www.image-net.org, accessed 25 January 2023) with a depth of 18 layers was repurposed and fine-tuned for the GNSS data in this article. The data were partitioned into 70% for training and 30% for testing. Cross-entropy was selected as the loss function and ADAM as the optimizer (The ResNet18 is configured as follows: pre-trained on *ImageNet*, propout = 0.5, learning rate = 0.001, optimizer = ADAM, loss function = CrossEntropyLoss).

Figure 11 shows the architecture of our ResNet. The input is a 64×3×125×193 image (i.e., a 193×125 RGB image), and the output is a 64×5 matrix.

### 5.4. Post-Processing

The post-processing component explores the robustness and indicates outliers in detection and classification. In general, our model outputs a prediction and its variance, i.e., the uncertainty, for every second over one minute. From there, a simple threshold-based decision method (on both the accuracy and the variance of all categories) selects the contextually correct interference class per minute and a naive moving average filter of ten time steps. It yields optimal results for detection (two categories) and classification (multiple categories). RF and ResNet provide mean values regarding the classification accuracy ($F_{\beta =2}$). However, to return the variance of these predictions, specific techniques are required, at least in the case of ResNet. A benefit of the RF is its ability to estimate the uncertainty for each prediction by utilizing out-of-bag (OOB) error estimates, i.e., RF implicitly returns a mean and a variance of its estimates as it incorporates the ensemble technique (forest of decision trees). Thus, the following techniques are employed to derive mean and variance from our ResNet model. In the post-processing step of the ResNet architecture, MCD is applied to the output layers to assess the uncertainty of each estimate.

With sparse data or a complex network (with many parameters), the model can memorize the training data (so-called overfitting to the data) and, as a result, work great with the data it saw during training, but gives poor results for unknown data [102]. Dropout addresses these problems, as it is a well-understood regularization technique to prevent overfitting. Dropout turns off a different set of neurons (with a predefined number of neurons) at each training step. Hence, with dropout, any information can disappear anytime during training. Therefore, a neuron cannot rely on just a few inputs: it must distribute the weights and consider all inputs. As a result, it becomes less sensitive to input changes, leading to a better generalization of the model.

In addition to regularization, dropout also provides a mean with an uncertainty estimate. As on each training iteration, dropout randomly selects the neurons to fail in each layer. According to the dropout rate of that layer, a different set of neurons is dropped each time. Thus, the architecture of the model is slightly different each time. Consequently, the result is an average ensemble of many different neural networks, each trained on just one batch of data. Hence, the results of the ML framework reflect the mean of an ensemble of the networks. From there, the variance (uncertainty) is derived if dropout is activated during interference.

The MCD model estimation can be computed as the average of *T* predictions:

(9) $$\begin{array}{cc} p & =\frac{1}{T}\sum_{i=0}^{T}f_{nn}^{d_{i}}\left(x\right) \end{array}$$

(10) $$\begin{array}{cc} c & =\frac{1}{T}\sum_{i=0}^{T}{\left[f_{nn}^{d_{i}}\left(x\right)-p\right]}^{2} \end{array}$$

where *p* represents the posterior predictive mean of the model output for a given input *x*. The equation computes the average of *T* forward passes through the model with dropout enabled, denoted as $f_{nn}^{d_{i}}\left(x\right)$, where $d_{i}$ is a dropout mask sampled from a Bernoulli distribution during each forward pass. By averaging the predictions across multiple dropout masks, the equation provides a more robust estimate of the model prediction. In the second equation, *c* represents the uncertainty of the model prediction for the same input *x*. The equation computes the average of the squared differences between each individual prediction and the posterior predictive mean computed in the first equation. This represents the variance of the model predictions, which can be used as a measure of uncertainty. The equation effectively quantifies the disagreement between the individual predictions and the mean prediction, providing a measure of how confident the model is in the prediction.

#### 5.4.1. Visualization

**Figure 14:**
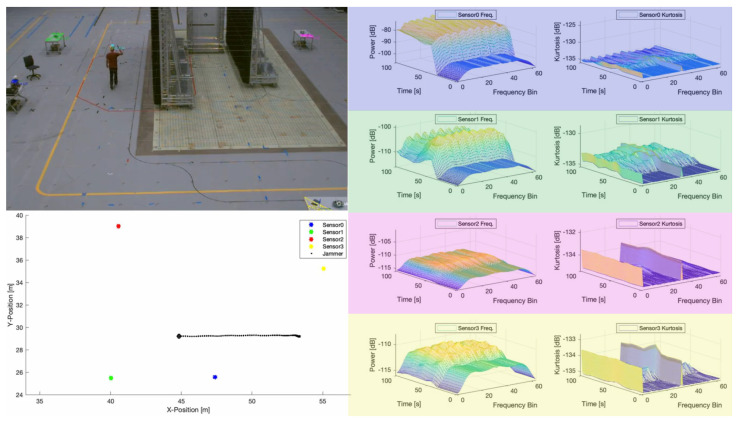
Exemplary GUI of the visualization component. Each sensor node is identified with a color (green, blue, pink, and yellow) in the photo in the top left, mapped to the ground plot in the bottom left, and the applicable data highlighted in the graphs in the right.

Figure 14 visualizes a scenario with a camera, reference data, and real-time spectrograms (a video of this test setup is also provided in the Supplementary Video S1 for this article). The Qualysis motion tracking system [60] provides both reference positions of all participants (see also the top view in the lower left corner) and an actual perspective for intuitive monitoring and visualization (the upper left corner). On the right, the GUI shows the synchronized feature streams of all sensor nodes in real-time. A person makes a trajectory around the blocking, reflective, and absorptive walls between listeners (Sensors 0 to 4) and carries a PPD transmitting an interference signal. Comparing the feature data streams, it is clear that the blue and green listeners show significant interference (significant increase in signal power). In contrast, the red listener is less affected (the walls block the line of sight). The signal strength of the yellow listener decreases as it increasingly loses sight of the interference signal.

## 6. Test Setup

**Figure 15:**
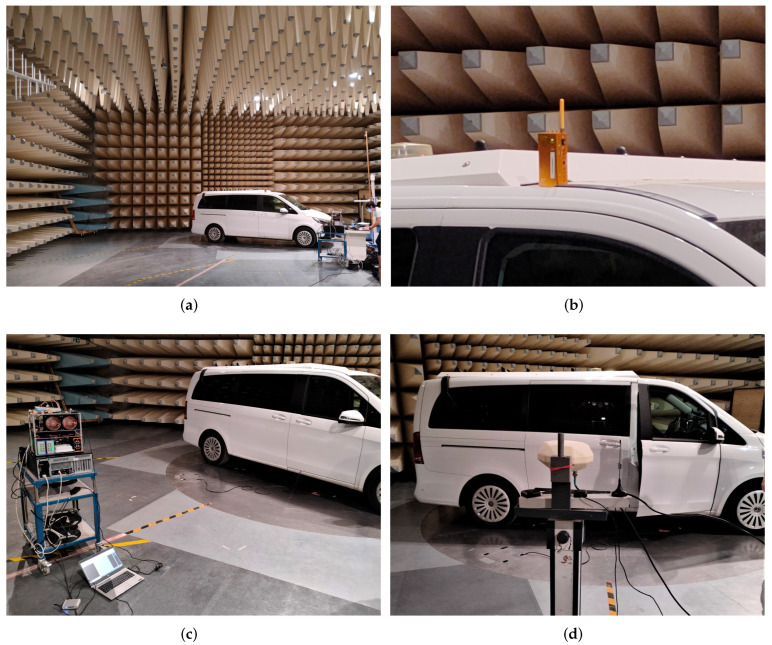
Test setup inside a large anechoic chamber, which is sufficiently big to fit a testing vehicle. Additionally, the vehicle is on a platform that can be rotated so that the signal path through different angles is measurable. (**a**) Inside the large anechoic chamber. (**b**) A PPD evaluated. (**c**) The monitoring system and other receivers (on the left). (**d**) Antenna mounting with different beam patterns.

Figure 15 shows some pictures of the measurement campaign. In the original conference paper, several results in an industrial measurement hall were conducted [36]. However, the results in this article are extended to an anechoic chamber to evaluate PPDs under realistic electromagnetic (EM) propagation through a vehicle. Therefore, this test case represents the application to detect and classify vehicular mounted interference commonly used for automatic toll collection (ATC). This case is challenging, as the PPD interferes significantly with the onboard navigation system, but the metal vehicle naturally shields the signal from an external observer. Furthermore, the vehicle results in different scattering profiles from different directions, which distorts the signal making classification more challenging.

In contrast to previous studies [62,66,67] that employed ML and DL methods for interference analysis, the framework is not only tested on synthetic data from laboratory simulations, but also in a deterministic real-world environment. Several experiments explore the limitations of the framework and the challenge of reliable detection and classification. Four common PPDs are chosen, each transmitting a chirp signal with a bandwidth ranging from 10 to 30 MHz.

The following experiments are performed:

- Exp-A: Static setup with one of the PPDs inside a driver cab of a van and a fixed distance of 6 m between the car and the sensor. The interference signals were activated sequentially. Only one at a time was transmitting.
- Exp-B: Static setup with one of the PPDs was placed inside the van at the driver’s side while the sensor was placed outside at distances of 3, 6, or 9 m from the vehicle. The experiment was conducted sequentially with all four interference signals, with only one interference signal transmitting at a time.
- Exp-C: To test complex real-world conditions and dynamic scenarios with moving interference, including line-of-sight (LOS), severe multipath, and non-line-of-sight (NLOS) conditions. This experiment was conducted at the L.I.N.K Halle Test Center at Fraunhofer Nürnberg. A person moved a COTS PPD across a tunnel of reflector wall, miming a typical motorway bridge in Germany. Four sensor nodes were mounted down each side of the bridge crossing.

**Figure 16:**
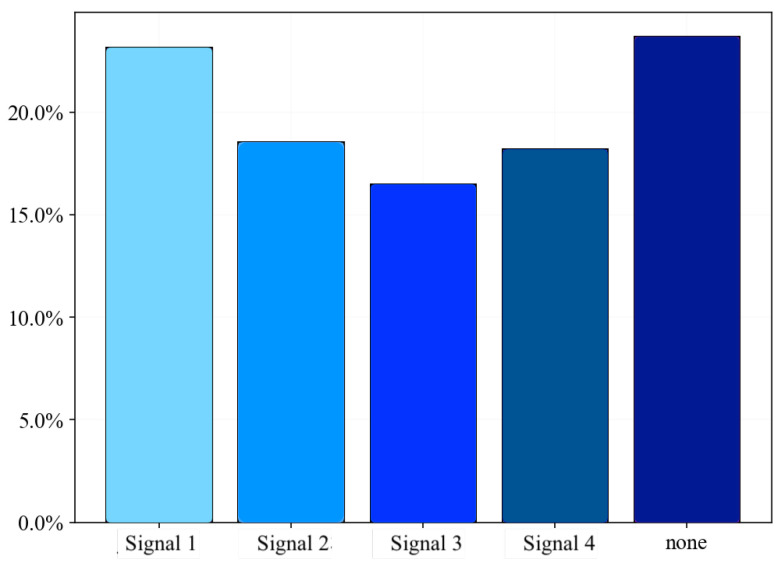
Example distribution of data from a single batch.

Each experiment included no interference, and the four mentioned interference signals facilitate detection and classification methods. During experiments Exp-A and Exp-B, which ran for more than 5 h, seven sensors collected more than 125,000 samples at 1 Hz. Figure 16 shows the distribution of the recorded data. The data set is divided into 70% training data and 30% validation or test data for each benchmark.

The original conference paper that this article extends [36], considered power scaling to shrink the effective detection distance down and to facilitate simple testing in a measurement hall. It indicates an approximate increase of factor 30 in the detection and classification range to what is tested. This approach is also used in Exp-C and demonstrates that detection and classification ranges for an equivalent PPD of 110 m is possible.

## 7. Results

### 7.1. Results—Exp-A

**Figure 17:**
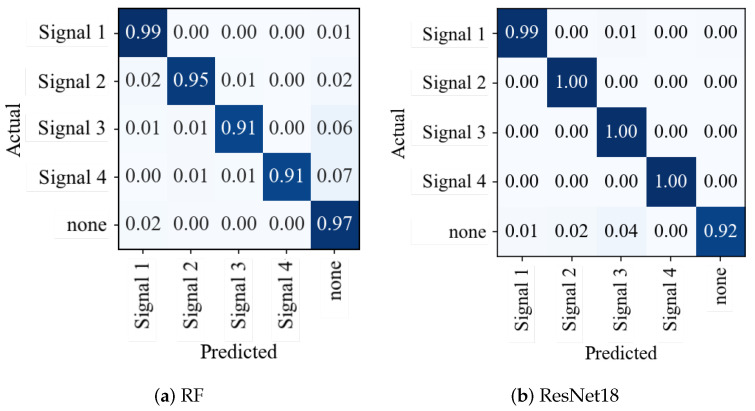
Comparison between confusion matrices of (**a**) RF and (**b**) ResNet. Darker colors indicate more allocation of the predicted and actual class combinations: ideally, the matrix should only have values on the diagonal.

In this experiment, four separate PPDs are placed inside a vehicle (see Figure 15) and are activated sequentially. A sensor node records the signals from 3 m to 9 m. Additionally, interference-free data are recorded. The RF and ResNet models were trained and tested on these data. The confusion matrices in Figure 17 show that the models were able to effectively differentiate between the four types of interference signal signals and the clean signal with an accuracy exceeding 94%.

#### RF vs. ResNet

The 64 SDR features with 5 s intervals are plotted as spectrograms and saved as PNG files (as shown in Figure 12) to compare the performance and accuracy of the RF and ResNet models. These files are then utilized for training a ResNet18 model, a deep CNN architecture with 18 layers (see Figure 11). The same 64 SDR features are used for an RF model with 1000 DTs (see Figure 13) to compare the accuracy. The overall accuracy of the ResNet18 model is marginally higher by approximately 1% compared to the RF model (see Figure 17). However, the ResNet18 model trains more than 100 times longer and requires more than 270 times longer for an inference step of a single sample (see Table 2).

### 7.2. Results—Exp-B

**Figure 18:**
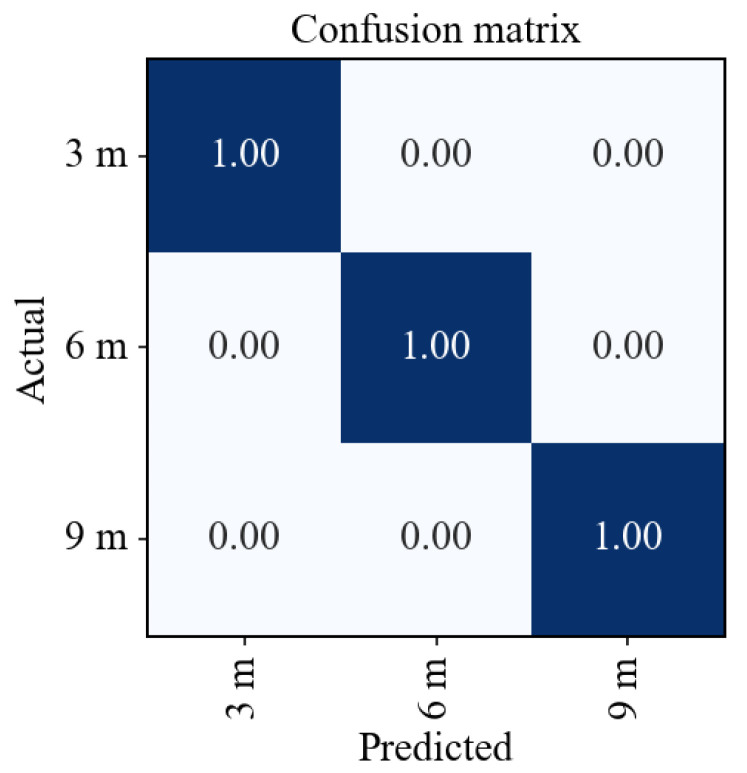
Confusion matrix of RF train on distances between sensor and interference signal.

In this experiment, four distinct interference signals were positioned inside a car and switched on sequentially. The sensor node was relocated to obtain different measurements, with distances of 3 m, 6 m, and 9 m between the sensor and the interference signals. An RF model with 1000 trees was trained on these data. It can classify the distances, as demonstrated in Figure 18.

The confusion matrix in Figure 18 shows that the RF model could accurately differentiate between the distances from the sensor to the interference signals, achieving a perfect accuracy of 100%. These results demonstrate that ranging is possible with ML. Therefore, they are a stepping stone to achieving localization with ML.

### 7.3. Results—Generalization

**Figure 19:**
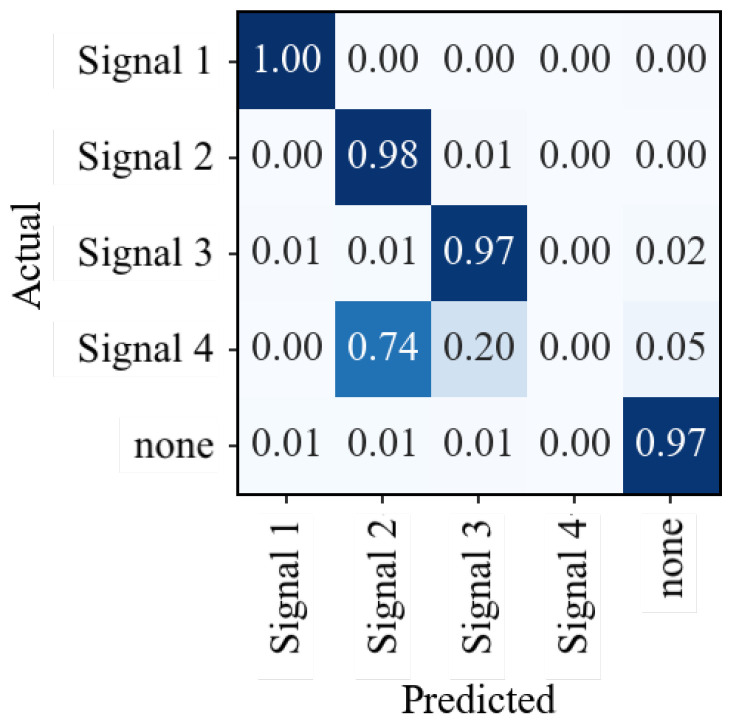
Generalization of model by removing the class “Signal 4” in the training data.

To test the generalization and robustness of new classes, an RF model was trained on three interference signal types, but tested on all four types, as in Exp-B. According to the confusion matrix presented in Figure 19, the misclassified instances of “Signal 4” are mainly assigned to the “Signal 2” category, with approximately 20% being classified as “Signal 3”. Only about 5% of the signals of “Signal 4” are assigned wrongly to the class “none”.

These results demonstrate that the RF model can correctly classify unknown interference signals, particularly those that share similarities with other known interference signals.

In summary, detection in unknown scenarios with NLOS components is significantly more challenging. However, the ML can successfully detect the interference signal in most cases.

### 7.4. Results—L.I.N.K.—Lane Detection

**Figure 20:**
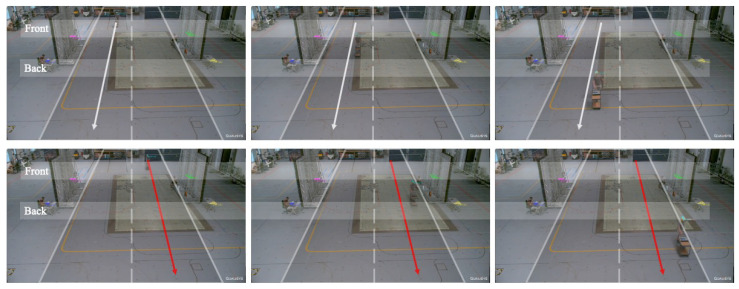
Lane detection experiment with four sensor nodes mounted on each side of a bridge crossing (two walls reflecting inside and absorbing outside) on a highway.

Results of experiment Exp-C extend experiment Exp-B and showcase the challenges of reliable detection and classification in dynamic scenarios. Figure 20 shows the setup of the lane-detection experiment.

This scenario mimics a typical motorway in Germany with two lanes going in the same direction. The lane detection experiment consists of four sensor nodes. Two are on the left (blue and purple sensors) of the highway, and two are on the right (yellow and green). They are symbolically mounted on each side of a bridge crossing. The “Front” and “Back” zones represent the entrance and exit zones of a typical tunnel of a highway bridge. In the “Front” zone, two sensor nodes are placed at the purple (left) and green (right) markers. In the “Back” zone, two sensor nodes are placed at the blue (left) and yellow (right) markers. The two walls reflecting inside and absorbing outside represent the reflection properties of the bridge.

The top row of photos in Figure 20 shows the trajectory (white arrow) of a vehicle with no interference along Lane A (left lane). The bottom row shows the trajectory (red arrow) of a vehicle carrying a PPD along Lane B (right lane). The sensor data streams (i.e., SDR vectors) received from the four sensor nodes are collected, synchronized, and used for lane classification. The data are pre-processed so that the sensor data are synchronized with the interference signal reference position. Hence, every position of the interference signal is known, and the corresponding signals are present at the four sensor stations at all times. From there, the sensor data are split based on the interference positions associated with a given lane. Thus, a dataset that contains two classes, Lane 1 and Lane 2, is created. From there, a classification method is formalized to determine the trajectory of the PPD. Additionally, it is possible to regress the absolute position in XY-coordinates on the dataset to determine the exact position of the PPD [43].

This experiment investigates how many (1 to 4) sensors are necessary to reliably classify the lane where an interference signal is driving. Table 3 shows the results of lane detection.

The results show that lane detection is improbable with a single sensor, but classification is possible. However, lane detection is achievable with two sensors (93.4%) and the classification is improved. The results indicate that adding more than three sensors adds too much (multipath) information that affects the localization performance. Two sensors (i.e., purple and green or blue and yellow, see Figure 20) on one side of the tunnel performed best. Instead, information from diagonal or three or more sensors reduces the lane detection accuracy. As fingerprint-based localization (detection) exploits the additional multipath information in the signal to map a unique fingerprint of the signal to a specific position, it is possible that adding too much information may not add additional information, but may result in either redundant information or even mirrors the information. It may confuse the model, i.e., the separation mechanism of the two lanes.

## 8. Conclusions

A low-cost COTS interference signal detection and classification platform, including detailed hardware, software, and algorithmic design description, is presented. It is significantly less expensive than comparable systems and dramatically reduces setup, calibration, and maintenance effort. Furthermore, the system demonstrates real-time pre-processing, visualization, detection, and classification with only a SBC.

Initial results show that accurate detection and classification are possible based on limited front end statistics and GNSS measurements. Tests in a measurement hall with severe multipath effects demonstrate successful classification despite NLOS or multipath reception. Further systematic tests in an anechoic chamber containing a full sizes van revealed that classification and distance estimation is possible, which paves the way for ML-based localization. Finally, tests in a measurement hall demonstrated that interference localization as a lane detection problem in high-way simulation is possible.

A systematic performance evaluation and extended real-world trials are planned for future research. Further, generalizing the models for reliable operation is emphasized.

In future work, the study of spectral features over time using temporal convolutional network (TCN) [103] and residual neural network (ResNet) [101] architectures and considering their model calibration and uncertainty estimation is proposed.

## Figures and Tables

**Table 1:** Power consumption test configurations and results.

No.	Description	Web Serv.	WiFi	ML	GNSS Log	SDR Proc	SDR Only	Mean [W]	Peak [W]
1	Full—debug	x	x	x	x	x	x	6.107	7.510
2	Full—op.		x	x	x	x	x	6.086	7.325
3	Full no link			x	x	x	x	5.692	7.145
4	External ML				x	x	x	5.593	6.932
5	Only GNSS				x			2.682	4.059
6	Only SDR					x	x	5.289	6.514
7	No SDR proc						x	3.692	4.137
8	Only RP							2.219	2.526

**Table 2:** Performance and training speed comparison between RF and ResNet classifiers.

AI Model	Training Time for the Whole Model *	Inference Time per Sample *	Accuracy	$F_{\beta =2}$ Score
RF (1000 trees)	**17.2 s** ${}^{†}$	**0.071 ms**	0.949	0.948
ResNet18 (32 epochs)	29:56 min = 1796 s	19.16 ms	**0.960**	**0.959**

* The training and the inference were performed on an *Apple M1 Max*, 10-core CPU, using tsai: 0.3.1, fastai: 2.7.9, and pytorch: 1.12.1. ^†^ The best values are shown in green and bold.

**Table 3:** Results of our lane detection experiment. The best value is shown in green and bold.

No. of Sensors	Interference Types	$F_{\beta =2}$ [%]	Var. [%]
1 (purple)		76.3	7.5
2 (front)		**93.4**	7.3
2 (back)	7 interference types	91.7	6.8
2 (purple, yellow)	with a total of 33 subclasses: Chirp,	87.1	4.8
2 (purple, blue)	Noise, Multitone, …	85.8	5.7
3 (2 front, blue)		81.9	7.2
4 (2 front, 2 back)		82.5	6.9

## Data Availability

Not applicable.

## Supplementary Materials

The following supporting information can be downloaded at: https://www.mdpi.com/article/10.3390/s23073452/s1.

## Abbreviations

The following abbreviations are used in this manuscript:

CN0	carrier-to-noise density ratio
ADS-B	automatic dependent surveillance-broadcast
AE	autoencoder
AGC	automatic gain control
AI	artificial intelligence
AOA	angle of arrival
API	application programming interface
ATC	automatic toll collection
CNN	convolutional neural network
COTS	commercial-off-the-shelf
CPU	central processing unit
DL	deep learning
DME	distance measurement equipment
DOP	dilution of precision
DSP	digital signal processor
DT	decision tree
DVB	digital video broadcasting
EM	electromagnetic
ES	electronic support
FDoOA	frequency difference of arrival
FFT	fast Fourier transform
FPGA	field-programmable gate array
GLRT	generalized likelihood ratio test
GNSS	global navigation satellite system
GPIO	general purpose input/output
GPSD	GPS service daemon
GPU	graphics processing unit
GUI	graphical user interface
HIL	human-in-the-loop
IQ	in-phase and quadrature-phase
ISR	interference-to-signal ratio
ISS	international space station
LEO	low earth orbit
LNA	low-noise amplifier
LOS	line-of-sight
LTE	Long-Term Evolution
MA	moving average
MCD	Monte Carlo dropout
ML	machine learning
NLOS	non-line-of-sight
NMEA	National Marine Electronics Association
OFDM	orthogonal frequency division multiplexing
OOB	out-of-bag
pHat	Raspberry Pi hat
PNG	portable network graphic
POI	probability of intercept
PPD	privacy protection device
PPS	pulse per second
PQF	polyphase quadrature filter
PRN	pseudo-random noise
PSD	power spectral density
PVT	position, velocity, and time
RAM	random access memory
ResNet	residual neural network
RF	random forest
RFFE	radio-frequency front-end
RHCP	right-hand circular polarized
RSS	received signal strength
SBC	single-board computer
SDR	software-defined radio
SSC	spectral separation coefficient
STFT	short-time Fourier transform
SVM	support vector machine
TCN	temporal convolutional network
TDOA	time difference of arrival
TPU	tensor processing unit
UART	universal asynchronous receiver-transmitter
